# Supplementary material for: Deep learning image recognition enables efficient genome editing in zebrafish by automated injections
Source: PLoS One. 2019 Jan 7;14(1):e0202377. doi: 10.1371/journal.pone.0202377 (PMC6322765; doi:10.1371/journal.pone.0202377)
Supplement: S1 Text — (DOCX) [file pone.0202377.s001.docx]

**Deep learning image recognition enables efficient genome editing in zebrafish by automated injections**

Maria Lorena Cordero-Maldonado^1¶,*^, Simon Perathoner^1¶,#a^, Kees-Jan van der Kolk^2¶^, Ralf Boland^3^, Ursula Heins-Marroquin^1^, Herman P. Spaink^3^, Annemarie H. Meijer^3^, Alexander D. Crawford^1,#b^, Jan de Sonneville^2,*^

# **S1 Text. Deep Learning Supplement.**

We created a deep learning system to predict if an injection should take place, and when a first cell is detected to determine the injection point. This injection point is annotated close to the first cell in early stage zebrafish eggs. The system consists of two consecutive inference steps.

First, a classification of the image into one of five categories is performed:

"empty" -- No egg is present in the image.

"inject" -- The first cell is visible.

"nocell" -- No cell is visible. It may be the case that one or more cells are positioned under the egg, and are thus occluded.

"twocell" -- More than one cell is visible. Typically, two cells are visible.

"sick" -- An egg is visible, but it is sick or damaged, or there is another anomaly.

One of the categories is labelled "inject", and signifies that a first cell is visible.

In case of "inject", the system performs a second inference step, in which the x,y position of a suitable injection point is determined (the depth of the injection is fixed).

Separate neural networks were used for both steps. For both networks, we used a modification of the Inception v3 architecture [Rethinking the Inception Architecture for Computer Vision ***Christian Szegedy, Vincent Vanhoucke, Sergey Ioffe, Jon Shlens, Zbigniew Wojna***; The IEEE Conference on Computer Vision and Pattern Recognition (CVPR), 2016, pp. 2818-2826].

Input images had the shape of 224 x 224 pixels. As a preparation step for training, validation and inference, we masked out parts of the image that are not of interest. Basically, we defined the circle C, inscribing the image boundary, and we blackened all pixels outside of C.

Our images were black and white. Since the Inception v3 architecture is defined for colour images, we simply pretend that our images are in colour. In the future, we might optimize the network specifically for monochrome images.

Classification

For the classification step, the neural network architecture consists of:

the top part of the Inception v3 network (containing all inception blocks);

a 2D global spatial average pooling layer;

a fully connected layer of 1024 nodes, with ReLU activation function;

a fully connected layer of 5 nodes, with softmax activation function

For training and validation we used the following numbers of images for each class:

| Label | #Training images | #Validation images |
| --- | --- | --- |
| Empty | 2472 | 581 |
| Inject | 2724 | 674 |
| Nocell | 2532 | 629 |
| Twocell | 1366 | 312 |
| Sick | 153 | 49 |

This data is not balanced. Class occurrences are, very roughly speaking, what can be expected when running the injection process.

Injection point determination

For the injection point determination, we need to map the neural network output to a (x,y) coordinate. We have taken the following approach. First, a triangular mesh is created, covering the coordinate space of the input image. We let the outputs of the neural net correspond to vertices in the mesh. In our case, we used 160 vertices.

To keep prediction as simple as possible, we postulate the following rule:

A minimal change in the coordinates (x,y) should result in a minimal change in the neural net output vector V, and vice versa.

We implement this rule by representing the point (x,y) using barycentric coordinates (a, b, c) in the mesh ABC that contains the point (x,y). The barycentric coordinates follow the normalizing relation

[http://mathworld.wolfram.com/BarycentricCoordinates.html]

a + b + c = 1.

Fig 1 shows some points (small red dots) with barycentric coordinates on a generic triangle ABC. Let (x_A_, y_A_), (x_B_, y_B_), (x_C_, y_C_) be the positions of the vertices of ABC. Then we can calculate the x and y position of any of the smaller red dots with the following formula:

x = a*x_A_ + b*x_B_ + c*x_C_

y = a*y_A_ + b*y_B_ + c*y_C_


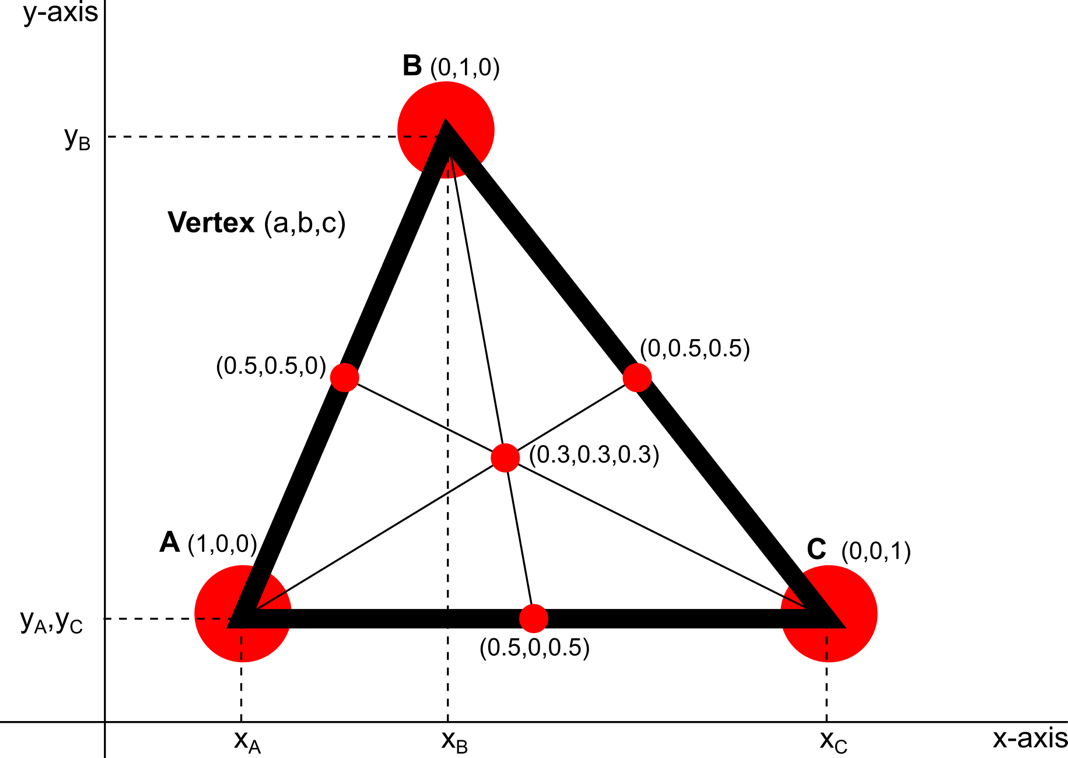


**Fig 1. Schematic view of the barycentric coordinate system.**

Note that these equations correspond to the centre-of-mass of three masses ‘a’, ‘b’, ‘c’, located at the vertices of the triangle. We say that barycentric coordinate ‘a’ corresponds to the vertex with coordinates (x_A_, y_A_); and similarly, for ‘b’ and ‘c’.

The relations can be inverted as follows:

a = ((y_B_ - y_C_)*(x - x_C_) + (x_C_ - x_B_)*(y - y_C_)) / ((y_B_ - y_C_)*(x_A_ - x_C_) + (x_C_ - x_B_)*(y_A_ - y_C_))

b = ((y_C_ - y_A_)*(x - x_C_) + (x_A_ - x_C_)*(y - y_C_)) / ((y_B_ - y_C_)*(x_A_ - x_C_) + (x_C_ - x_B_)*(y_A_ - y_C_))

c = 1 - a - b

These equations are valid as long as the triangle ABC is not degenerate (i.e., not all vertices on the same line).

For training, we use the second set of equations, whereas for inference, we use the first set of equations.

Although one triangle could cover all possible injection positions within an image, a finer mesh is used to improve the accuracy of the algorithm. To see how this would work in practice we extend the triangle, and we look at point P, see Fig 2.


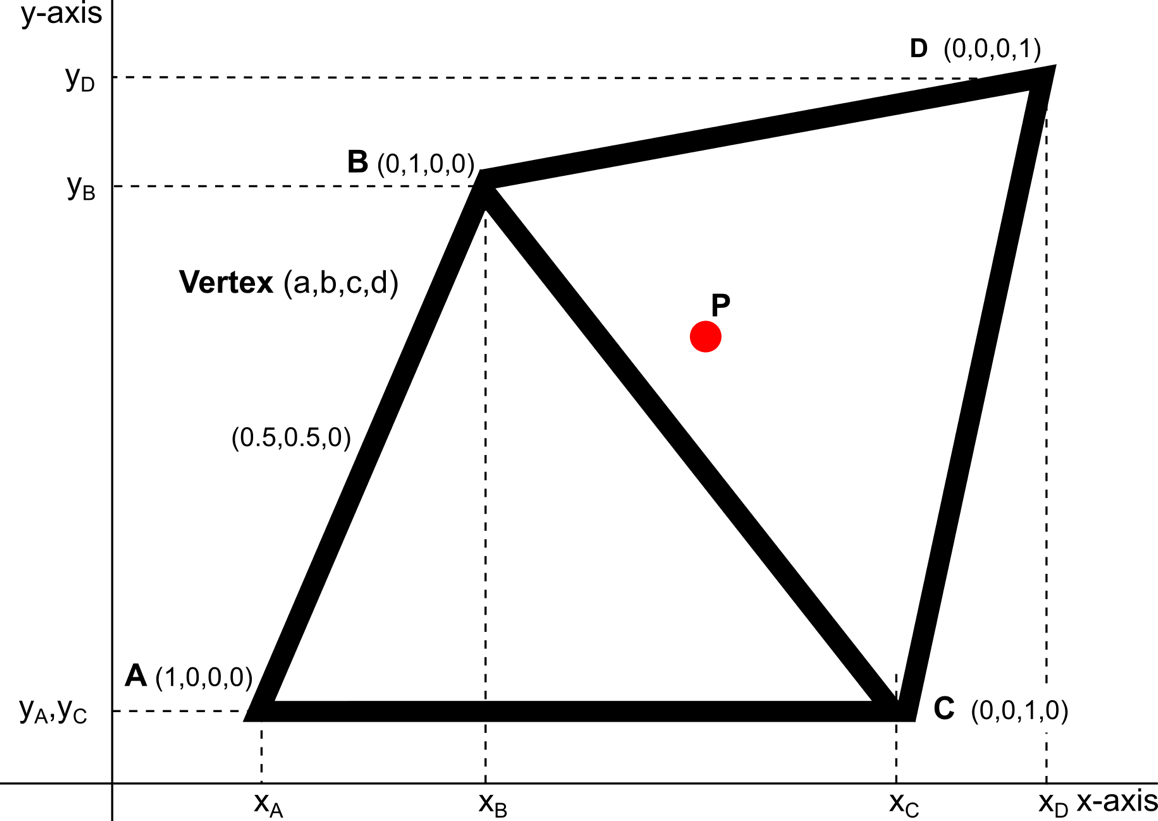


**Fig 2. Schematic view of a mesh of two triangles and a point P, located in the triangle on the right.**

The output of the neural net will result in a vector with values between 0 and 1 for each vertex. So, for this mesh the expected output for P is something like (0,0.45,0.30,0.25). However, the neural network is not normalised to generate centre of mass coordinates that sum up to 1. Therefore, a possible output for P would be (0.02, 0.6, 0.45, 0.3). Then, to obtain the (x, y) position of P, normalisation has to be included in the formula to obtain Cartesian coordinates. To obtain x and y for an output vector V, we generalize the first set of equations using the centre-of-mass analogy. The equations become:

x = (v_i_ * x_i_)/(Sum v_i_)

y = (v_i_ * y_i_)/(Sum v_i_)

For P(0.02, 0.6, 0.45, 0.3), for x, this would result in:

x = (0.02*x_A_+0.6*x_B_+0.45*x_C_+0.3*x_D_) / (0.02+0.6+0.45+0.3)

Note that the triangle covering a given point does not need to be unique, since the point may lie on an edge of the mesh. However, by using barycentric coordinates, the actual triangle that we use does not matter, since the barycentric coordinates corresponding to opposing vertices will be zero in that case. It is also easy to see that by crossing an edge or a vertex, the barycentric coordinates do not change abruptly.

The mesh used was created with the following considerations:

the triangles should be "far" from being degenerate;

the mesh should be "regular" and sufficiently dense in the area of interest, i.e., the centre circle of the image (where the egg can be found). The mesh is depicted in Fig 3. This mesh was created by a simple force-directed scheme that is not detailed here further.

**Fig 3. Schematic view of the mesh used to find the injection position.**

The neural network architecture consists of:

the top part of the Inception v3 network (containing all inception blocks);

a 2D global spatial average pooling layer;

a fully connected layer of 1024 nodes, with ReLU activation function;

a fully connected layer of 160 nodes, with softmax activation function

We used 2724 images for training and 674 images for validation (these are the same images as used for label "inject" in the classification step).

Training

To prevent overfitting, it is generally necessary to use as many training samples as possible. Our training set was quite large, but for the chosen architecture still caused overfitting. Therefore, we artificially increased the number of training samples by performing four types of image transformation:

rotations about the center of the image;

zooming by a factor 0.9 ... 1.1;

shifting by 28 pixels orthogonally in the +/- x and y direction;

flipping the image horizontally

We performed these transformations for training and validation. For consistency, we also performed these transformations for inference. These transformations could also be used to make multiple predictions for a single image, and to perform postprocessing on the results, e.g., averaging and removing outliers. We did not exploit this possibility because increasing the number of predictions would require more processing time, and this does not seem justified by the small improvement in accuracy.

Note that for the training phase of the injection point determination, the injection point was transformed (x,y) along with the image. For the inference phase, the inverse transformation was performed.

Training of the classification step was done using the Adam stochastic optimizer [D. Kingma and J. Ba. **Adam: A method for stochastic optimization.** ICLR, 2015.], with a learning rate of 10^-4^. A plot of the training and validation accuracy is shown in Fig 4.

**Fig 4. Graph depicting the training history of the classification.**

Training of the injection point determination step was done using Adam, with a learning rate varying from 10^-3^ to 10^-5^. A plot of the training and validation accuracy is shown in Fig 5.

In both cases we used a loss function based on the categorical cross-entropy.

On our hardware, training took on the order of a working day (8 hours) for both steps.

Note that for the injection point determination, validation accuracy does not exceed 66%. However, the definition of accuracy we use here is based on classification, and in this case, there are 160 classes, each corresponding to a vertex in the mesh. Thus, we are predicting the vertex nearest to the injection point. This is a more difficult problem to solve than predicting the injection point directly, since the injection point may often lie at roughly equal distance from three vertices. Hence, we should not be concerned too much about the reduced accuracy here. Instead, we make a different graph that shows a histogram of the distance between the predicted and annotated injection point. This graph is shown in Fig 3 in the main article.

**Fig 5. Graph depicting the training history of the injection position.**

Software and hardware used:

Keras 1.2.2

Theano 0.9.0

NumPy 1.11.0

SciPy 0.17.0

Shuttle SZ170R8 equipped with:

CPU: Intel Core i3 6100

Memory: 16 GB kit Kingston DDR4 2133Mhz, ECC

GPU: NVidia GeForce GTX 1070
