# Supplementary material for: Deep learning image recognition enables efficient genome editing in zebrafish by automated injections
Source: PLoS One. 2019 Jan 7;14(1):e0202377. doi: 10.1371/journal.pone.0202377 (PMC6322765; doi:10.1371/journal.pone.0202377)
Supplement: S1 File — (DOCX) [file pone.0202377.s002.docx]

**Deep learning image recognition enables efficient genome editing in zebrafish by automated injections**

Maria Lorena Cordero-Maldonado^1¶,*^, Simon Perathoner^1¶,#a^, Kees-Jan van der Kolk^2¶^, Ralf Boland^3^, Ursula Heins-Marroquin^1^, Herman P. Spaink^3^, Annemarie H. Meijer^3^, Alexander D. Crawford^1,#b^, Jan de Sonneville^2,*^

# **S1 File. Movie demonstrating robotic injections with deep learning.**

<http://www.lifesciencemethods.com/Deep_learning_based_microinjection.mp4>
