# Supplementary figures and images for: Deep learning image recognition enables efficient genome editing in zebrafish by automated injections
Source: PLoS One. 2019 Jan 7;14(1):e0202377. doi: 10.1371/journal.pone.0202377 (PMC6322765; doi:10.1371/journal.pone.0202377)

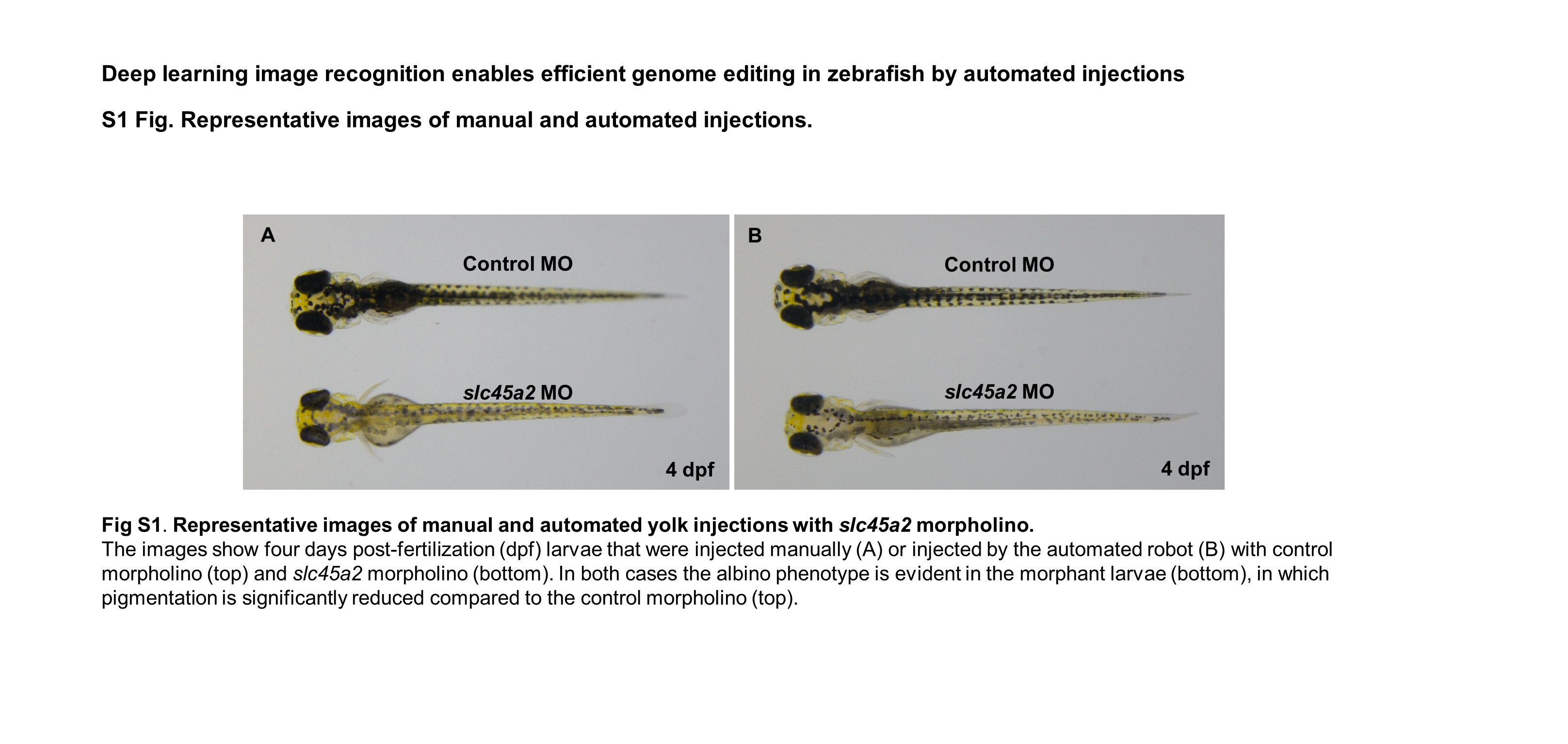

Supplement: S1 Fig — The images show four days post-fertilization (dpf) larvae that were injected manually (A) or injected by the automated robot (B) with control morpholino (top) and slc45a2 morpholino (bottom). In both cases the albino phenotype is evident in the morphant larvae (bottom), in which pigmentation is significantly reduced compared to the control morpholino (top). (TIF) [file pone.0202377.s005.tif]

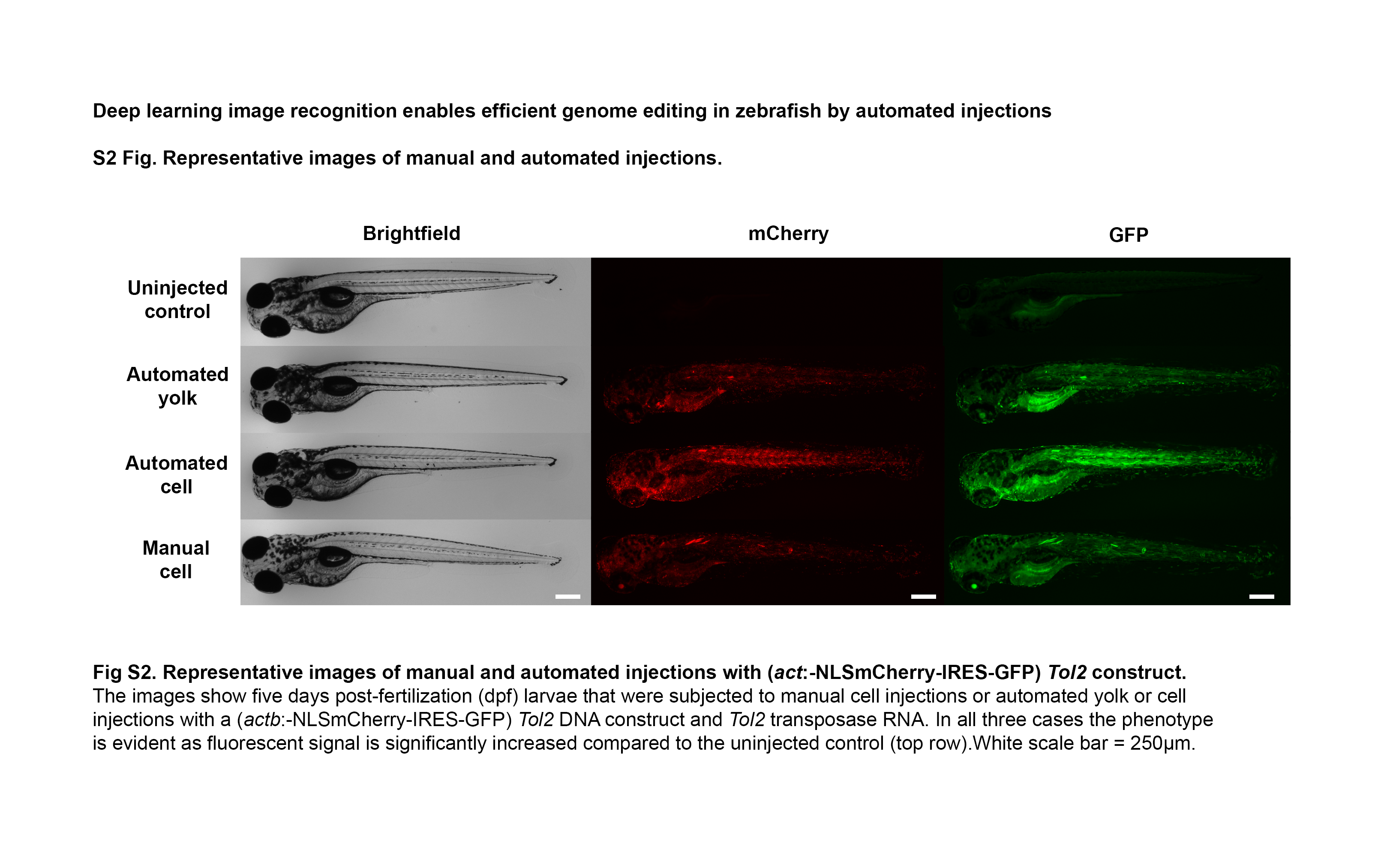

Supplement: S2 Fig — The images show five days post-fertilization (dpf) larvae that were subjected to manual cell injections or automated yolk or cell injections with a (act:-NLSmCherry-IRES-GFP) Tol2 DNA construct and Tol2 transposase RNA. In all three cases the phenotype is evident as fluorescent signal is significantly increased compared to the uninjected control (top row). White scale bar = 250μm. (TIF) [file pone.0202377.s006.tif]

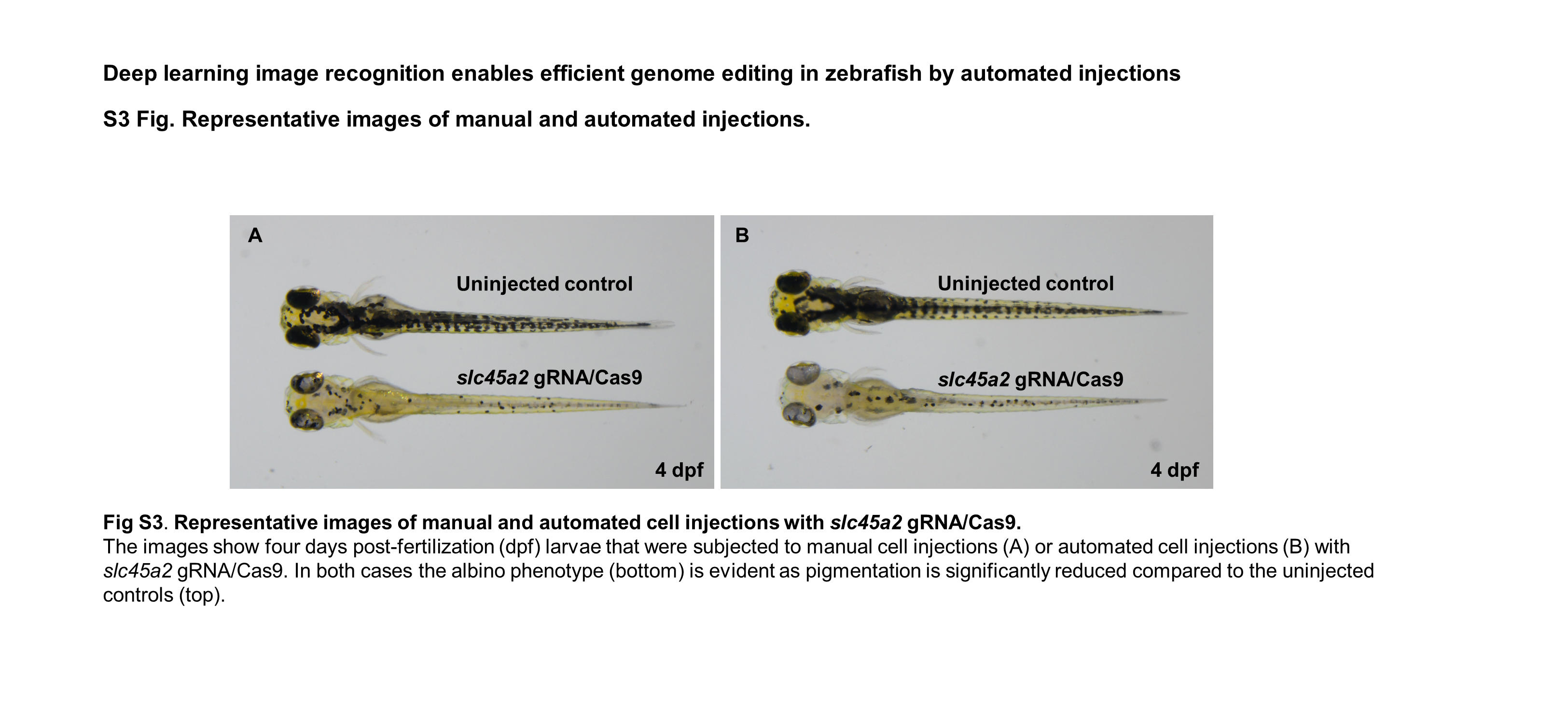

Supplement: S3 Fig — The images show four days post-fertilization (dpf) larvae that were subjected to manual cell injections (A) or automated cell injections (B) with slc45a2 gRNA/Cas9. In both cases the albino phenotype (bottom) is evident as pigmentation is significantly reduced compared to the uninjected controls (top). (TIF) [file pone.0202377.s007.tif]
